# Exploring Phytochemical Profile, Pharmaceutical Activities, and Medicinal and Nutritional Value of Wild Edible Plants in Ethiopia

**DOI:** 10.1155/2024/6408892

**Published:** 2024-07-24

**Authors:** Woinshet Kassie Alemu, Limenew Abate Worku, Rakesh Kumar Bachheti, Archana Bachheti, Adam Mekonnen Engida

**Affiliations:** ^1^ Department of Industrial Chemistry College of Natural and Applied Sciences Addis Ababa Sciences and Technology University, P.O. Box-16417, Addis Ababa, Ethiopia; ^2^ Department of Chemistry College of Natural and Computational Science Debre Tabor University, Debre Tabor, Ethiopia; ^3^ Department of Allied Sciences Graphic Era Hill University, Society Area, Clement Town 248002, Dehradun, Uttarakhand, India; ^4^ Department of Environment Science Graphic Era (Deemed to Be University) 248002, Dehradun, Uttarakhand, India

**Keywords:** medicinal use, nutritional value, pharmaceutical activities, phytochemical profile, wild edible plants

## Abstract

In many parts of the world, wild edible plants (WEPs) constitute an essential component of the global food basket, providing an alternative source of wholesome and nourishing food. Ethiopia is one of countries of the world having largest concentrations of WEPs. In the country, various parts of WEPs, such as fruits, stems, leaves, tubers, roots, or entire plant sections, are frequently consumed and used as food sources for famine relief during seasonal food shortages, as well as for commercial purposes. WEPs have been also used in the country as sources of phytochemicals, traditional medicine, and pharmaceutical applications. Approximately 30%–40% of WEPs and over 413 different kinds of WEPs are commonly consumed by Ethiopians regularly. Most plant families utilized as WEPs are Moraceae, Fabaceae, Flacourtiaceae, Myrtaceae, Rosaceae, and Tiliaceae. The most widely used plant parts of WEPs were fruits. WEPs can be used as substitutes for traditional plant-based human diets because of their high nutritional value, which includes proteins, vitamins B2 and C, and low moisture content. This review focuses on using edible wild plants for pharmacological purposes, dietary supplements, and alternative medicine. Many obstacles prevent people from consuming WEPs, even when they are easily accessible and available. The use of WEPs must be encouraged by nutrition policies as one of the pillars of food and nutrition security. To increase yield, diversify the revenue streams of small-scale farmers, and protect the diminishing wild edible fruit resources, it is imperative to domesticate and enhance WEPs.

## 1. Introduction

Food and nutrition security is one of our planet's most significant issues. It is estimated that two billion people are undernourished, which increases their susceptibility to illness. This can significantly hinder economic growth [1]. Food security is a serious issue, especially in sub-Saharan African countries that depend largely on imports [2]. Nonetheless, the region is home to various biodiverse habitats that support valuable, wild, and edible plants that are used as food sources and cannot be domesticated or farmed [3].

Numerous types of wild edible plants (WEPs) can mitigate the global food problem on a worldwide scale. According to the most recent Food and Agriculture Organization (FAO) 2019 State of the World Report on Biodiversity for Food and Agriculture, 15 out of the 91 nations that submitted data regularly use wild foods in their national diets. The taste of staple foods is enhanced by using WEPs, which almost one billion people worldwide use to supplement their diets with extra nutrients [4]. WEPs are an essential component of the global food basket in every region of the world and one of the substitute sources of wholesome food. These naturally occurring edible plants have been crucial in helping impoverished communities in many rural areas of the world meet their food and nutritional needs and improve their general health [5]. Countries such as Indonesia, Papua New Guinea, and Sri Lanka are known to use WEPs for food and medicine in a moderate manner. Still, countries like India, Malaysia, Nepal, and the Philippines use it extensively [6]. Ethiopia is one of the several nations in the sub-Saharan Africa region with abundant WEP resources, which have been exploited to maintain and sustain human life [7].

Ethiopia is one of Africa's nations with the highest concentrations of WEPs. Ethiopia's diverse flora provides a wealth of wild and semiwild plants that can be eaten for their fruits, stalks, leaves, tubers, roots, or entire plant sections [8]. Hundreds of WEPs are reported to be occasionally consumed by rural communities in several underdeveloped nations, such as Ethiopia [9]. Ethiopian rural people rely on WEPs to help them survive, particularly during famine, dry spells, and other disasters and emergencies [5]. Ethiopia communicates that about 30%–40% of its people regularly eat wild plant species [10]. According to Lulekal et al. [4], Ethiopians consume roughly 413 types of WEPs divided into 224 genera and 77 families. According to a research done in the Quara District of northwest Ethiopia by Tebkew et al. [11], almost 70% of WEPs were used to supplement staple foods and fill in food shortages caused by famine and drought. According to Amente [12], a different researcher, the majority of the indigenous Gumuz people in Kamash Woreda periodically view WEPs as famine foods or meals to be consumed when starving. About 73.33% of the WEPs in the study area were utilized as extra food, and the remaining 26.67% were used as meals or as regular food.

Phytochemical substances are what give WEPs their nutritional worth. They contain various phytochemical substances, including phenolic compounds, terpenoids, and alkaloids, which may offer users several health advantages [13]. These plant-based phytochemicals contain antioxidants that can strengthen defenses against several illnesses, including diabetes, cancer, heart disease, neurological disorders, and aging [14]. Oxidation processes can produce reactive oxygen species, such as oxygen free radicals, which set off a series of events that could produce undesirable byproducts or cell damage that could cause various illnesses [15]. Antioxidants from WEPs are, therefore, vital to human health. These molecules, which guard against diseases like cancer, rheumatoid arthritis, diabetes, and other conditions, are not produced by the human body and must be obtained through diet [16]. By scavenging free radicals and preventing the oxidation of other biomolecules, phytochemicals like polyphenols and other bioactive substances can stop chain reactions [17, 18]. According to Chipurura, Muchuweti, and Kasiyamhuru [19], a single vegetable or plant food item has been found to contain over 900 distinct phytochemicals. Numerous phytochemicals are antioxidants, helping shield cells from reactive oxygen species–induced oxidative damage [17, 18]. Edible wild plants with high vitamins or carotenoids also have strong antioxidant properties. Numerous substances, including vitamins C and E, carotenoids, chlorophyll derivatives, alkaloids, flavonoids, phenolic acids, and other phenolic compounds found in higher plants, are linked to antioxidant qualities [20].

Historically, WEPs have given local populations all throughout the world financial support. However, these traditional uses still need to be recalled because of the loss of local awareness and knowledge as well as the modern way of life [21]. Thus, as these species are a possible solution for global food security, proper authentication, scientific analysis for nutritional composition, toxicity, phytochemical ingredients, and traditional knowledge of WEP must be recorded. This review is aimed at documenting and informing communities about WEPs' nutritional potential and phytochemical constituents. It also seeks to identify alternative food sources for the expanding population and address malnutrition and food insecurity issues requiring prompt attention and action. Furthermore, to identify current research gaps, this work proposes to describe the existing data on the nutritional potentials and phytochemical status of some edible wild plants in the nation.

## 2. Material and Methods

Publicly available documents served as the review's source materials. All of the material on WEPs in Ethiopia that the author has included comes from reputable sources, including Mendeley, Web of Science, Scopus, and Google Scholar. After evaluating all accessible Ethiopian ethnobotanical papers, information on Ethiopian WEPs, including their scientific names, nutritional and medicinal qualities, phytochemistry, and consumer perception, was acquired and compiled. A comprehensive review of more than 140 publications was conducted after collecting various scientific sources based on topic and academic field criteria. The results are included in the reference list. The collected data is analyzed critically and organized methodically based on its intended applications. Descriptive statistics were employed in an Excel spreadsheet to analyze the data and determine the quantity and proportion of frequently used plant components and the mode of consumption, marketability, and therapeutic value of whole WEPs.

## 3. WEPs as Sources of Food

WEPs provide for rural populations, substitute essential foods during food shortfall or shortage, and maybe a source of dietary supplements to prevent malnutrition [22]. Approximately one billion individuals eat wild foods (mainly plant-based) regularly worldwide [23]; according to Belcher, Ruiz-Pérez, and Achdiawan [24], wild forests provide a significant portion of the livelihood for nearly 300 million people.

**Figure 1:**
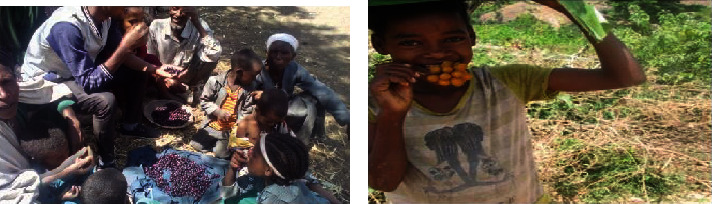
Fresh fruit of (a) *S. guineense* and (b) *C. africana* eaten by the people in the study area.

Research showed that, despite differences in age, sex, time of day, and season, Ethiopia's rural residents have a greater understanding of, tradition with, and opportunity to use WEPs [9]. They have distinct contributions in numerous ways since they are a vital part of the diet of many rural people [25]. In Ethiopia, people have used edible wild plants when food was scarce. They are also eaten to cover nutritional deficits and enhance staple dishes (Figure 1) [5]. Lulekal et al. [4] noted about 413 WEPs consumed in Ethiopia. They can contribute to the food resources in the home in emergency, seasonal, and supplemental ways. But locations with food insecurity are where consuming is more prevalent [26, 27]. For instance, the recurrent weather shocks that hinder agricultural productivity and cause food shortages appear to have worsened the woredas WEPs in southern Ethiopia, specifically in Konso, Derashe, and Burji [26]. According to research by Tebkew and Atinkut [28] in the Quara District in northwest Ethiopia, people ate WEPs to make up for food shortages caused by famine and drought (about 35%) and to augment staple foods (about 70%). Additionally, a study revealed that WEPs offer impoverished populations an additional source of monetary revenue [29]. The selection of these edible plants is based on simplicity of processing, good taste, time of availability, and low labour requirement [30]. According to Amente [12], most of the indigenous Gumuz people in Kamash Woreda periodically view WEPs as food for famine or starvation. About 73.33% of the WEPs in the study area were utilized as extra food, and the remaining 26.67% were used as meals or as regular food. WEPs are essential in household food security and nutrition in certain rural locations, especially those with dry soils. They can be used as an emergency food source during a famine, to augment staple foods, and to bridge seasonal food shortages [31, 32]. According to Assefa and Abebe [30], 15 (or 50%) of the 30 identified wild edible tree and shrub species are used to supplement the regular food supply. In the isolated regions of southern Ethiopia, several WEPs, including *J. ladanoides*, *C. grandis*, *T. foenum-graecum*, *A. graecizans*, *C. argentea*, and *P. quadrifida*, are ingested to help combat malnutrition and contribute to food security [9]. According to a study by Guzo, Lulekal, and Nemomissa [33] of fifty underutilized WEPs from 30 families and 39 genera, the families Moraceae, Fabaceae, Flacourtiaceae, Myrtaceae, Rosaceae, and Tiliaceae have the most significant number of species used as WEPs. Only the indigenous population consumes several underappreciated wild edible fruits found in the East Wollega Zone, such as *G. erubescens*, *F. mucuso*, *D. abyssinica*, and *Z. spina-christi* [34]. The Meinit cultural community in Bench Maji Zone, Southwest Ethiopia, grows and eats WEPs including *S. nigrum*, *V. membranacea*, *D. praehensilis*, *T. madagascariense*, and *C. gynandra* [35]. Plants such as *Z. spina-christi* fruit, *C. africana*, and *B. aegyptiaca* were used as WEPs in Ethiopia's northern and rift valley regions [36]. Northern Ethiopians often consume WEPs such as *A. digitata*, *B. aegyptiaca*, *C. africana*, *C. spinarum*, *T. indica*, *X. americana*, and *Z. spina-christi* [37]. Fruit eating by children from plant species includes *Ficus* spp., *R. abyssinica*, and *C. edulis* [37]. In South Gondar Zone, northwestern Ethiopia, fresh fruit of *S. guineense* is eaten by the people within the community (Figure 1) [38]. *A. hybridus*, *E. arabicum*, *E. abyssinicum*, *H. scimperii*, *H. rueppelii*, *R. nervosus*, and *U. simensis* were the most commonly used and well-liked WEPs [39]. A few researches have been done on WEPs in Ethiopia [5, 40].

The most commonly used edible wild plant parts were fruits, leaves, and gum. The fruits, seeds, and leaves of wild edible trees and shrubs are the components that are eaten [41]. According to Anbessa [42], fruits accounted for 79.31% of all edible plant parts used. The remaining edible plant parts were tubers and fruits (3.45%), young shoots (6.90%), young shoots and fruits (3.45%), roots (3.45%), and gums (3.45%). Since preparing fruit does not require much time, the use of wild fruits is higher than that of other plant parts, suggesting that the plants are more frequently utilized during food scarcity. Hungry people gather food from its natural habitat and consume it immediately, without waiting for preparation or cooking. This clarifies why the communities are well informed about the rise of wild fruit trees [43]. However, the findings of Mesfin, Demissew, and Teklehaymanot [44] and Amenu [45] showed that roots are mainly utilized for edible and medicinal uses.

Conversely, Guyu and Muluneh [46] found that wild vegetables accounted for 52.4% of all wild foods, around five times more than fruits and roots. According to another study, fruits are the plant parts most frequently employed for medical and edible reasons among various sections of wild and medicinal edible plants [12]. According to Leta's [47] research, of the 51 WEP species, roughly 40 were fruitful, 7 were leafy, and 2 were each of roots and flower nectars; the remaining 3 were made up of seeds, young shoots, and stem bark. The differences in how different plant species adapt to various ecological zones and local human cultures may account for the variety in the consumption of edible wild plant parts [40].

**Figure 2:**
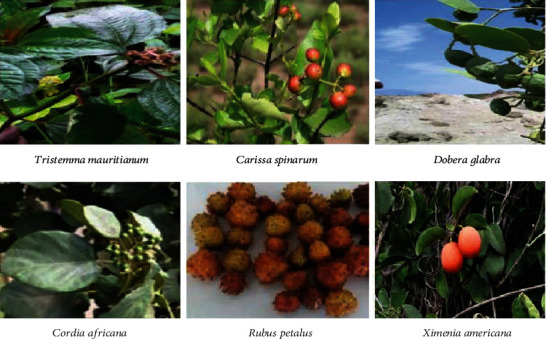
List of commonly consumed wild edible fruits.

Figure 2 shows that the most commonly reported for utilization include *U. simensis*, *C. africana*, *X. americana*, *T. indica*, *S. guineense*, *D. abyssinica*, *F. sur*, *F. vasta*, *P. peruviana*, *R. abyssinica*, *R. steudneri*, *C. spinarum*, *M. stenopetala*, *Opuntia ficus-indica*, and *S. nigrum* [10, 27, 48].

Most research investigations revealed that the gathered WEPs were consumed in their freshest forms. In Bullen District, Northwest Ethiopia, the consumption patterns of WEPs indicate that 57.1% are eaten raw, 16.9% are boiled, 6.5% are consumed in juice form, 9.1% are boiled or eaten raw, and 5.2% are consumed as porridge or sauce [40]. According to the study by Beche, Gebeyehu, and Feyisa [49], most edible species were eaten raw, without ripening or processing. According to a study done in the Quara District in Northwest Ethiopia by Tebkew et al. [11], people ate WEPs raw, dried, boiled, or prepared in various ways. Just nine of the edible plants were eaten after they had dried; the majorities were eaten while still fresh. The majority of WEPs in Bule Hora Woreda, southern Ethiopia, were reported by the locals to be consumed raw (approximately 89.66%); most of the edible sections of these plants did not require boiling, roasting, or other preparation before eating [42]. According to Ashagre, Asfaw, and Kelbessa [50], the majority of plant parts (87%) were consumed raw or uncooked, while a small percentage (6.5%) required processing and boiling to make them palatable. A smaller percentage (6.5%) could be consumed either way. It is possible to cook, boil, or ferment *A. caudatus*, *D. bulbifera* L., and *P. schimperi* for consumption [42].

The taste for WEPs varies; certain WEPs are only eaten during times of famine and are not eaten at other times [40]. Each person's preference for consuming edible wild plants differs depending on the location [47]. Every plant has a different condition or consumption window. Certain plants are constantly eaten, even when there is a sizable food supply, while other plants are only eaten during severe food shortages and scarcity [51]. According to Tahir et al. [52], the Mieso people use wild plants; they only eat *P. montanus*, *V. apiculata*, *R. natalensis*, *P. capensis*, *M. undata*, *A. prasinata*, *C. monoica*, *E. racemosa*, *M. kummel*, *Z. spina-christi*, *M. africana*, *H. micranthus*, and *S. americanum* during famines or times of food scarcity. During food scarcity, famine WEPs are crucial to rural communities' survival [53]. According to Feyssa et al. [54], all-season WEPs such as *B. aegyptiaca*, *Z. spina-christi*, *X. americana*, and *Grewia* spp. were identified; they produced fruits throughout short and dry rain seasons and grew abundantly during dry seasons. *G. tenax* produces badheesa, or “hungry plant,” fruits from April to June. In Oromo, this plant is also referred to as beela baalesa. When agriculturally farmed crops are scarce, *G. schweinfurthii* produces fruits that are fit for human eating, even by adults. During the green famine, *A. alboviolaceum*, *A. bombycina*, *B. prestinaria*, *B. scleroneura*, *C. rubens*, *C. caesiu*, *J. ladanoides*, and *L. nepetifolia* were used in the southernmost part of Benishangul-Gumuz regional state in western Ethiopia [46].  Table 1 shows widely used WEP species in different geographical location of Ethiopia and their suitable months to eat them.

## 4. WEPs as Nutritional Value

Despite their little role in family meals, wild food plants have the potential to be significant cultural and nutritional resources for indigenous populations worldwide. Numerous sources claim that the nutrients in wild edible foods are either higher or equivalent to those in cultivated plants [59]. WEPs are nutrient-rich, including vitamins like E and C, lipids, phenols, minerals, carbs, proteins, and dietary fiber. They also have a high-calorie value [60].

### 4.1. Proximate Composition of WEPs

Analyses of the proximate composition of WEPs are very important to predict their suitability for consumption. Yiblet and Adamu [61] found that the leaves of *A. graecizans* had a maximum moisture content of 40.8 ± 0.00 g/100 g and an ash content of 24.70 ± 0.15 g/100 g. Their findings also revealed that the fruits of *O. cusindica* had 44.4 ± 0.00 g/100 g of carbohydrates, while the shoots of *R. abyssinicus* had 14.07 ± 0.03 g/100 g of crude fat and 34.70 ± 0.25 g/100 g of fiber. These indicated that the plants are important WEPs for consumption. According to another findings from prior studies, the highest lip was found in WEPs called *V. paradoxa*, and the highest ascorbic acid concentration was found in *T. indica* and *P. edulis* [62]. The fruit pulp of *A. digitata* contains significant fat, protein, carbs, and vitamin C [63]. *C. grandis* discovered a 36.3% protein content [9]. The findings demonstrated that compared to cultivated vegetable crops such as *B. oleracea* (2.7 g/100 g), *B. carinata* (2.8 g/100 g), and *A. sativum* (4.5 g/100 g), these wild edible fruits have comparatively higher protein values [9]. According to Jiru, Fekadu Gemede, and Keyata [34], the crude protein content of wild edible fruits included *G. erubescens*, *F. mucuso*, *D. abyssinica*, and *Z. spina-christi* ranged from 3.01% to 5.31%, the crude fat content from 1.40% to 3.31%, and the crude fiber content from 0.71% to 2.11%. The WEP samples analyzed by Mokria et al. [36] revealed that *C. africana*, *B. aegyptiaca*, *Z. spina-christi*, and *B. aegyptiaca* had significant crude protein levels. WEPs have varying moisture contents: 5.9 g/100 g for *E. sativa* leaves and 12.9 g/100 g for *R. sativus* roots [64]. The fruit of *T. madagascariense* had the highest fat content among the five plants studied by Yimer et al. [35], including *S. nigrum*, *V. membranacea*, *D. praehensilis*, *T. madagascariense*, and *C. gynandra*. This fruit could be employed as an ingredient in producing high-energy food products. On the other hand, the *D. praehensilis* tuber's low fat content suggested that it might aid in the reduction of obesity and cardiovascular disease. The WEPs, *S. nigrum*, *V. membranacea*, *D. praehensilis*, *T. madagascariense*, and *C. gynandra*, have crude protein contents ranging from 4% to 21.7% [35]. *D. praehensilis* tuber had the lowest total protein content, whereas *S. nigrum* leaves had the highest protein content. After the fruit of *T. madagascariense*, the tuber of *D. praehensilis* had the highest carbohydrate content. Similar findings were reported by Adamu et al. [39], who discovered that in northeastern Ethiopia, the carbohydrate content of WEPs ranges from 30.7 g/100 g in *C. argentea* to 60.5 g/100 g in *P. laticoronum*. On a dry weight (DW) basis, the moisture content of the *A. hybridus* seed and the *U. simensis* leaf was 9.17 g/100 g and 9.77 g/100 g, respectively. The lowest moisture content is found in *H. schimperi* and *H. rueppelii* leaves, at 6.50 and 6.95 g/100 g, respectively. *E. abyssinicum* (33.63 g/100 g), *U. simensis* (30.55 g/100 g), and *E. arabicum* (30.15 g/100 g) were found to have relatively high protein concentrations, while *H. rueppelii* and *Amaranthus hybridus* leaf had the lowest, at 13.1 g/100 g and 17.63 g/100 g, respectively [39]. Similar findings were observed for *C. grandis* and *A. gomboczianus* in southern Ethiopia, where the protein contents were 36.3 and 5.8 g/100 g, respectively [65]. According to the data on protein content, those that consume WEPs at the locations where they grow depend on the WEPs with the highest protein content. Where children's protein intake is lowest, such as in some sections of the Amhara Region, these plant categories may help increase protein intake [66]. The bulk of the wild edible fruits under study had protein values that were comparatively greater than those of cultivated vegetable crops, including *A. sativum* (1–4.5 g/100 g), *B. oleracea* (1.1–2.7 g/100 g), and *B. carinata* (2.5–2.8 g/100 g) [9].  Table 2 shows the proximate composition analysis of most well-known WEPs for checking their suitability for consumption.

### 4.2. Mineral Composition of WEP

Different WEPs have different mineral composition depending on type of species, growing climate condition, and type of soil [68]. According to studies by Adamu et al. [39], the magnesium concentration of WEPs in *U. simensis* leaves ranges from 72.79 mg/100 g to 56.65 mg/100. In another research finding, in the Lasta District, the potassium (K) content of *H. schimperi* leaves ranges from 54.30 mg/100 g to 14.40 mg/100 g in seed of *A. hybridus*. Regarding to the calcium content (Ca) of WEPs, 44.35 mg/100 g calcium was found in the leaves of *E. arabicum* and higher amount of calcium (60.14 mg/100 g) was found in the leaves of *U. simensis*. In the Benishangul-Gumuz Region of Ethiopia, the Ca levels of WEPs in this study are higher than those of *D. abyssinica* (43.19 mg/100 g) and *O. abyssinica* (24.49 mg/100 g) WEPs [69]. According to research by Uyoh, Ita, and Nwofia [65], the content of iron (Fe) was 10.51 mg/100 g DW in *R. nervosus* young shoots and 27.96 mg/100 g DW in *H. schimperi* leaves. In southern Ethiopia, *L. intybacea* had a Fe concentration of 22.0 mg/100 g, while *X. caffra* had a 1.9 mg/100 g value. The figure is higher than *C. esculenta*'s Fe concentration (10.57 mg/100 g) in the Wolaita Zone of Ethiopia [70]. According to Getachew et al. [9], green leafy vegetables contributed a value of 6177 mg% for the Ca content of *J. ladanoides*. Fe, Mg, Mn, and Zn contents in the same plant varied from 11.7 to 23.14, 175 to 2049, 3.4 to 9.9, and 1.2 to 3.3 mg%, respectively. Jiru, Fekadu Gemede, and Keyata [34] discovered that *F. mucosa* had a high Ca and phosphorus (P) content, whereas *Z. spina*-christi fruit had high Fe, zinc, and magnesium content. The study by Mokria et al. [36] on WEP samples revealed that *B. aegyptiaca* and *Z. spina-christi* had greater Ca values. The K, Fe, and zinc concentrations of *B. aegyptiaca* and *C. africana* were approximately 50% higher than those of *Z. spina-christi*. The powdered leaves of *V. membranacea* had the lowest sodium (Na) content (174.9 mg/100 g), greatest Fe (38.5), and copper (Cu) (0.5) contents. Furthermore, *T. madagascariense* fruit powder had the lowest concentration of minerals, including Cu (0.1 mg/100 g), Zn (2.4 mg/100 g), and Fe (0.8 mg/100 g). On the other hand, Yimer et al. [35] discovered that *C. gynandra* leaf had low Cu (0.1 mg/100 g) values despite having high amounts of various mineral elements, including Na (277.4 mg/100 g), K (1487.8 mg/100 g), Ca (594.8 mg/100 g), and Mg (588.1 mg/100 g). According to Yiblet and Adamu's [61] research, Ca was found in *U. simensis* leaves in significant amounts (754.9 ± 0.23 mg/100 g), followed by Fe (31.63 ± 0.03 mg/100 g) and Zn (3.09 ± 0.02 mg/100 g) in immature *R. abyssinicus* shoots.  Table 3 shows the presence of some important metallic elements such as Ca, Na, Fe, K, Zn, and Mg in different WEPs with various concentrations. The above data showed that the majority of wild edible fruits studied offer relatively higher protein contents than cultivated vegetable crops such as *B. oleracea* (1.1–2.7 g/100 g), *B. carinata* (2.5–2.8 g/100 g), and *A. sativum* (1–4.5 g/100 g) [9]. The work of Yimer et al. [35] showed that cultivated edible plants contain larger amount of macro- and micronutrients, comparable to WEPs. Woldegiorgis et al. [71] performed proximate composition analyses on wild mushrooms including *A. campestris*, *L. sulphureus*, *T. clypeatus*, *T. microcarpus*, *T. aurantiacus*, *T. microcarpus*, *T. letestui*, and *Termitomyces* spp. as well as cultivated edible mushrooms like *P. ostreatus*, *L. edodes*, *A. bisporus*, and *A. bisporus*. The findings indicated that, in comparison to cultivated edible plants, the highest protein and fat contents were found in the WEPs of *A. campestris* and *T. microcarpus,* with a value of 36.7 ± 0.08 and 5.16 ± 0.08, respectively. In the same study, compared to cultivated food plants, WEPs including *T. microcarpus*, *A. campestris*, and *L. sulphureus* had the highest ash, fiber, and carbohydrate contents (25.3 ± 0.12, 11.9 ± 0.64, and 82.3 ± 0.45, respectively).

## 5. Commercial Value of WEPs in Ethiopia

Numerous edible wild fruits can be found in nearby markets, serving as a significant revenue stream [30, 73]. A household's financial capacity to purchase food from the market is essential to the food access pillar [74]. You can sell or trade edible wild fruits, leaves, juice, and regional beverages to earn money and find work. For impoverished households, revenue from selling wild plant species is significant [30]. Additionally, WEPs might help make money by selling to their internal market or exporting to nearby nations, primarily Sudan [55]. Balemie and Kebebew [10] assert that the revenue generated from the sale of wild plants holds significant value for impoverished households since they need to augment food production with financial resources to fulfill their fundamental necessities. Numerous commercially viable wild fruits have also significantly boosted the production of revenue. Marketable wild edible fruit species include those of *A. digitata*, *B. aegyptiaca*, *C. africana*, *R. abyssinica*, and *Z. spina-christi* found in northern part of Ethiopia [37, 75]. In addition, other marketable plants such as *A. senegalensis*, *B. rotundifolia*, *F. indica*, *M. tetraphylla*, and *S. birrea* are found in southern Ethiopia [30, 37, 76], and *B. discolor*, *C. spinarum*, *D. mespiliformis*, *M. kummel*, *S. guineense*, *T. indica*, *X. americana*, and *Z. spina-christi* are found in northwestern part of Ethiopia [11, 37, 75]. The fruits of *S. guineense*, *B. aegyptiaca*, *B. neglecta*, and *X. americana*, as well as the leaves of *M. stenopetala*, are among the WEP parts that women and children most frequently sell and offer a way to augment household income [47]. The sale of WEPs supplements low agricultural yields and adds extra income to households, according to studies done in Ethiopia [51]. According to research on the marketability of WEPs, most WEPs (75.7%) in East Shewa were not commercialized. About 24.3% were discovered to be extensively marketed from the wild harvest to the neighborhood market [54]. While the remaining species were marketed for food uses, species including *T. indica* L., *Z. spina-christi*, *C. spinarum* L., and *H. abyssinica* were sold for their medicinal use. Similarly, investigations carried out elsewhere in Ethiopia revealed that the fruits of species including *T. indica*, *C. spinarum*, and *M. kummel* were commercially viable WEPs [10, 48, 55]. These plants can offer clues regarding commercially valuable flora resources connected to local knowledge. Furthermore, responsible commercial exploitation is critical to further developing and enhancing the use of underutilized wild food plants [77]. With a few exceptions, the WEPs in southern Ethiopia's Bule Hora Woreda are hardly ever offered for sale in the local market as food. Specifically, only *S. guineense* has historically been offered for sale during food scarcity in the region. But these foraging edible wild plants are not very popular these days. Food scarcity does not exist in the study area, but the WEP *Tamarindus* is available in the local market [42]. People have been observed to consume fruits of *B. aegyptiaca*, *Z. spina-christi*, *X. americana*, *B. discolor*, *G. flavescens*, *G. tenax*, *G. villosa*, *G. schweinfurthii*, and *O. ficus indica* in the East Shewa Zone of Ethiopia, according to a market evaluation [54]. Research conducted in the northwest Ethiopian province of Chilga has revealed that WEPs with commercial potential include *C. spinarum*, *C. olitorius*, *D. mespiliformis*, *D. abyssinica*, *F. sur*, *H. cannabinus*, *M. kummel*, *S. comorensis*, *S. guineense*, *T. indica*, *X. americana*, and *Z. spina-christi* [55]. The result of the research indicated that *T. indica* and *H. cannabinus* were the most highly priced species.

Several social, economic, and cultural aspects contribute to the marginal revenue gained by the sales of EWFTSs. This result implies that to improve the commercialization of EWFTSs, future promotion campaigns should be created with packages that include social and cultural suits tailored to particular local contexts [78].

## 6. WEPs as Phytochemical Sources in Ethiopia

The naturally occurring chemical substances that the body produces as a result of regular metabolism and which affect how well it uses nutrients are called phytochemicals. Phytochemicals diminish the nutritional value of food as they decrease the bioavailability of dietary components, specifically protein, minerals, and vitamins [35]. Researchers showed that WEPs such as *A. graecizans*, *O. ficus-indica*, *R. abyssinicus*, and *U. simensis* were found to have phytochemicals including phenols, alkaloids, tannins, triterpenes, saponins, and caconoids in their fruit [61]. Yiblet and Adamu [61] found that the leaves of *A. graecizans* and the fruits of *O. ficus-indica* contained alkaloids and phenols, respectively. In the same study, phytochemicals such as phenols, alkaloids, tannins, triterpenes, saponins, and caconoids were found in *A. graecizans*, *O. ficus-indica*, *R. abyssinicus*, and *U. simensis*.

A study showed that water-soluble polyphenol compounds such as tannins are found in five different WEPs such as *S. nigrum, V. membranacea, D. praehensilis, T. madagascariense, and C. gynandra*. The highest tannin contents are found in the leaves of C. gynandra (329.0 mg/100 g) while the lowest was found in D. praehensilis tuber (5.8 mg/100 g). A study conducted on northern part of Ethiopia showed that total phenolic content of WEPs such as *A. hybridus*, *E. abyssinicum*, *E. arabicum*, *H. rueppelii*, *H. schimperi*, *R. nervosus*, and *U. simensis* were 10.00, 13.13, 12.69, 8.62, 17.02, 11.69, 16.28, and 0.79 mg GAE/100 g, respectively [79]. In the work of Adamu et al. [79], leaves of WEPs such as *E. abyssinicum* and *E. arabicum* contain phenolic content of 12.69 ± 0.00 mg GAE/100 g and 8.62 ± 0.02 mg GAE/100 g, respectively. This value is lower than that of two cultivated edible plants such as *C. gynandra* and *A. caudatus* (257 mg GAE/100 g) [24]. The TPCs of the underutilized wild edible fruits such as *G. erubescent*, *F. mucus*, *D. abyssinica*, and *Z. spina-christi* were 230.76, 191.61, 191.36, and 108.32 mg GAE/100 g, respectively [34]. This value is higher than the total phenolic content of *M. indica* with value of 83.2 mg/g, 115.8 mg/g, and 79.6 mg/g for hexane, ethanol, and petroleum ether extracts, respectively [80]. Methanol extract of nutritional cultivated plant in Ethiopia called *M. stenopetala* had the lowest total phenolic (39 ± 3 mg of GAE per gram of dried extract) contents than the above WEPs [81].

Total flavonoid content (TFC) in methanolic extracts for *D. praehensilis* tuber and *C. gynandra* leaf varied from 0.85 to 11.25 mg catechin equivalent per gram (mg CE/g). Jiru, Fekadu Gemede, and Keyata [34] discovered that the fruit of *G. erubescens* has high content of total flavonoid (112.85 mg CE/g) and phenolic (230.76 mg/GAE/g). In other study, WEPs like *A. hybridus*, *E. abyssinicum*, *E. arabicum*, *H. rueppelii*, *H. schimperi*, *R. nervosus*, and *U. simensis* contain TFC of 5.97, 7.12, 6.23, 3.57, 5.43, 5.74, 2.27, and 5.02 mg QE/100 g, respectively [79]. A study conducted in the East Wollega Zone, Western Ethiopia, on the TFC of underutilized wild edible fruits, revealed that WEPs, namely, *G. erubescens*, *F. mucuso*, *D. abyssinica*, and *Z. spina-christi*, had TFC values of 112.85, 88.10, 91.51, and 79.70 mg GAE/100 g, respectively [34]. Methanol extract of *M. stenopetala* which is cultivated in Ethiopia had lowest total flavonoid (11 ± 2 mg CE/g of dried extract) contents than the above WEPs [81]. In the work of Forsido, Rupasinghe, and Astatkie [82], the total content of cultivated plant *E. ventricosum* was found to be 55.9 (mg GAE/100 g dry extract). This value is still lower than WEPs mentioned above.

In other studies, the total oxalate content of WEPs varied from 43.7 in *T. madagascariense* fruits to 443.9 mg/100 g in *S. nigrum* leaves. Compared to cultivated vegetables, the study's oxalate levels were lower, ranging from 189.12 mg in *L. sativa* lettuce to 630.4 mg/100 g in *A. esculentus* okra [83]. However, total oxalates ranged from 14.34 mg/100 g in *B. lycium* to 362.66 mg/100 g in *N. officinale*, which is a little higher than that reported by Shad, Shah, and Bakht [84]. Research revealed that compared to cultivated plants, wild plants produced greater levels of antioxidant components, including vitamins C and E [20]. Compared to many cultivated species, WEPs frequently have higher nutritional and bioactive chemical contents. Comparing *U. simensis* to other popularly grown and consumed green leafy vegetables in Ethiopia, it was discovered that *U. simensis* had a higher nutritional content [9]. The ascorbic acid content of raw *U. simensis* leaves was also higher than that of *S. oleracea* (32 g/100 g), *L. sativa* (6 g/100 g), *B. vulgaris* (18 g/100 g), and *B. carinata* (2 mg/100 g) [20].

The nutritional makeup and phytochemical content of edible wild plants, such *A. abyssinicus*, were examined in the research by Tsehay et al. [85]; the findings indicated that the plant contained several phytochemicals, including flavonoids (4.93 ± 0.03 mg/100 g of gallic acid), total phenol (107.63 ± 0.04 mg/100 g of gallic acid), oxalate (344.56 ± 1.64 mg/100 g), and tannins (0.15 ± 0.05 mg/100 g of catechin). In another study determination of phytochemicals, *X. caffra* fruits contain tannin content of 6314 mg of gallic acid [9]. In the work of Adamu et al. [79], the tannin concentrations of the leaf extracts of *E. abyssinicum*, *E. arabicum*, *H. rueppelii*, *H. schimperi*, and *U. simensis* were 3.73, 5.49, 1.38, 2.60, and 2.21 mg/100 g of catechin. The highest tannin content is found in the leaves of *E. arabicum* (5.49 mg/100 g), followed by in the young shoots of *R. nervosus* (9.09 mg/100 g) and in the leaves of *H. schimperi* (3.60 mg/100 g). The least tannin content (1.38 mg/100 g) is determined from the leaves of *H. rueppelii* [79]. The tannin content of the WEPs tested is lower than that of *C. esculenta* (243.06 mg/100 g) cultivated in Ethiopia [86] and from the tannin content of triticale (285.56 mg/100 g) reported from Amhara Region, Ethiopia [87].

## 7. WEPs Used as Traditional Medicine

The knowledge and procedures employed in traditional medicine are utilized to diagnose, prevent, and treat medical issues. It only relies on firsthand knowledge and observations that are verbally and in writing transmitted from generation to generation [88]. Approximately 64% of people depend on traditional medicine to meet their medical needs [89]. It is well recognized that traditional medicine is used in many African, Asian, and Latin American nations to address some of their primary healthcare needs [90]. In Ethiopia, there are 6000 species of higher plants, and around 14% are utilized as traditional medicines [91].

WEPs are widely used in many nations as everyday foods, functional foods, nutritional supplements, and medicinal plants. Their main goal is to promote health [92]. Wild plants are excellent candidates for traditional medicinal use because they contain various plant secondary metabolic products, including polyphenols, terpenoids, and polysaccharides [93]. According to a study done in Lebanon, most human diseases can be cured by eating wild foods [94]. Research conducted in Ethiopia's Konso ethnic community revealed that various edible wild plants could treat various illnesses [8]. Eighteen kinds of WEPs identified in the research area of Ethiopia's Nech Sar National Park are used by the local community as traditional medicines and food sources, according to Leta [47]. According to locals in Ethiopia's Amhara Region, the most widely utilized edible and nutraceutical plants are *A. africanus*, *C. africana*, *C. spinarum*, and *X. americana* [95]. Their findings showed that of the WEPs utilized for traditional medicinal purposes in the area, shrubs (50%), herbs (21%), and trees (29%) were the most common. According to Addis, Asfaw, and Woldu [8], fruits of WEPS, including *T. indica*, *R. natalensis*, *C. africana*, and *B. aegyptiacus*, are used medicinally. There are references to the fruit of *C. africana*, which is used to alleviate diarrhea. Abdominal pain can be relieved with the leaves of *S. nigrum*, while tapeworm can be treated with the roots of *C. spinarum* [25, 27]. In Southwest Ethiopia, WEPs including *S. nigrum*, *V. membranacea*, *D. praehensilis*, *T. madagascariense*, and *C. gynandra* are commonly utilized for a variety of traditional medicinal and culinary purposes [35]. *B. aegyptiaca* and *Acacia etbaica* WEPs are used to treat and manage skin infections and anthrax, respectively, in the Raya-Azebo district of the Tigray Region, Northern Ethiopia [96]. In Mieso District, WEPs are also used medicinally. For example, the fruit of *T. indica* was used to treat scabies, intestinal parasites, gastritis, and nausea. *Z. mucronata* was used to treat snake bites, *Z. spina-christi* was used to treat dandruff and skin diseases, *C. monoica* was used to treat itching, *M. africana* was used to treat intestinal parasites, *B. aegyptiaca* was used to treat bloating, and five species such as *F. indica*, *G. ferruginea*, *H. abyssinica*, *B. salicina*, and *R. natalensis* were used to treat cancer [52]. The local community uses the raw leaves of *F. carica* to treat hemorrhoids. At the same time, its fruit is consumed raw to enhance vision, and its latex is applied to pimples and eruptions, blood purifier, miswak, and skin issues [97].

## 8. Pharmaceutical Activities of WEPs

### 8.1. Antioxidant Activities

Plant sources are rich in polyphenols, flavonoids, vitamin C, carotenoids, tannins, and proanthocyanins; these phytochemicals shield the body from free radicals and are natural antioxidants [98]. WEPs are one of these plant sources that include nutritional and bioactive components that are yet to be discovered and may be able to avert oxidative stress [99]. As shown in Table 4, different phytochemicals such as carotenoids, flavonoids, phenols, vitamin C, and tannins which are responsible for antioxidant activities are present in different parts of WEPs [58].

Several academics have studied edible wild plants as possible sources of antioxidants. Of all the known WEPs with antioxidant qualities found in Ethiopian flora, Adamu et al. [79] reported the antioxidant activity of *A. hybridus* and *R. nervosus* leaves. According to the results, 50% of the free radical scavenging activities were found to have values of DPPH and FRAP that were less than 50 *μ*g/mL. According to the investigation, *A. hybridus* and *R. nervosus* have stronger antioxidant activity than usual crops. Because of the presence of some bioactive substances such polyphenols, flavonoids, flavonols, proteins, tannins, oxalates, phenolics, and micronutrient concentrations, the leaves of *A. graecizans* and *A. viridis* demonstrated antioxidant properties [100]. A study conducted by Engelhardt et al. [101], the antioxidant properties of *Amaranthus* species are caused by the presence of significant compounds such caffeic acid and quercetin-3-rutinoside (rutin). Bioactive compounds such as rutin from *C. abyssinica* leaf extracts, flavan-3-ol-7-O-glucoside from *H. johannis* root extracts, and 7-O-methylaloeresin A from *A. harlana* leaf latex were the most potent compounds to give plants high IC50 values of 3.53, 0.19, and 0.014 *μ*g/mL, respectively [102]. When the antioxidant activity of the WEPs was tested in vitro using DPPH assays, *S. nigrum* showed the maximum DPPH inhibition (87.63%), followed by *C. gynandra* (81.48%), *T. madagascariense* (80.41%), and *V. membranacea* (72.18%) [58]. However, DPPH inhibition in all sample extracts was significantly lower than the standard ascorbic acid (97.44%). Similar findings were reported by Lulekal et al. [4] and Yimer et al. [35] who found that the DPPH inhibition varied between 28.56% and 72.6% in the ethanol extract of *G. carpinifolia* and between 50.01% and 73.66% in the methanolic extracts of *H. sabdariffa*. According to a study, leaves of *R. nervosus* and *A. hybridus* that were taken from Ethiopian plants showed antioxidant activity, the greatest DPPH and FRAP values demonstrate in both plants [79]. Studies conducted in North Gondar, in the Amhara Region of Ethiopia, showed that the fruit and seed of *S. guineense* had TPC, EC_50_, and AA contents ranging from 581.25 to 1917.40 mg GAE/100 g, 4.02 to 155.7 mg/100 g, and 1.96 to 0.94 mg/mL, respectively. This indicates that the plant possesses high antioxidant qualities [103]. The assessed DPPH free radical scavenging capacity of the WEPs extracts was computed by comparing them to a 200 *μ*g/mL ascorbic acid standard. The *R. nervosus* extract of young shoots showed a higher percentage of inhibition (97.30%) than *A. hybridus* (leaf) (92.89), *E. abyssinicum* (leaf) (84.99), *E. arabicum* (leaf) (83.02), *H. schimperi* (leaf), and *A. hybridus* (grain) (52.31%) [79]. The study also showed that the FRAP assay of *H. schimperi* leaf extract increased at greater concentrations, producing a higher FRAP assay result (529.82 mM) at 300 *μ*g/mL. In the same study, finding also indicated that *A. hybridus* leaf extract has antioxidant potential with FRAP assay value of 485.45 mM. However, at 300 *μ*g/mL, the *A. hybridus* seed extract yielded the lowest FRAP assay result 139.25 mM compared to leaf extract [104].

### 8.2. Anti-Inflammatory

*O. lamiifolium* has been utilized in Ethiopian traditional medicine to treat a variety of inflammatory conditions, including fever, sore throat, wounds, and discomfort. Both the aqueous and ethanol extracts showed a significant reduction in inflammation; however, at all dose levels, the aqueous extract showed stronger anti-inflammatory action [124]. At doses of 300 mg/kg and 500 mg/kg body weight, hydroalcoholic extracts of plants that are used in Ethiopia to treat skin conditions were tested for their anti-inflammatory properties in mouse paw oedema caused by carrageenan*. Malva verticillata* and *Syzygium guineense* were the sources of the extracts, and both extracts demonstrated oedema inhibition at doses of 300 and 500 mg/kg [125].

### 8.3. Anticancer Activities

Medical plants provide cultural, ecological, and economic purposes and are vital to applying medical methods for a wide range of illnesses. They have been utilized as alternative medicine worldwide for over 2000 years [126]. About 80% of these therapeutic plants are used in medicine worldwide to prevent infections and cure disease [127]. Over the years, medicinal plants have been used consistently to treat cancer, especially in most underdeveloped nations worldwide [128]. Medicinal plants include phytochemicals or bioactive compounds that treat various illnesses, including cancer [129]. For instance, over 60% of the medications required for the cancer treatment system are naturally occurring plant-based products [130]. A study on traditional medicinal plants in Ethiopia found that malignancies and tumors, stomachaches, wounds, coughs, headaches, skin conditions, toothaches, and diarrhea were among the common ailments these plants were said to heal [131]. Studies conducted on the ethnobotanical use of MPs in different regions of the nation have revealed that traditional medicinal plants are frequently used to treat various cancer conditions, including skin, breast, and lung cancer [126]. Cancer patients prefer traditional medicinal herbs over conformist therapy approaches due to their accessibility and cultural acceptance [132]. Very few WEPs are used for the treatment of cancer.  Table 5 gives some wild plant fruits in Ethiopia used to treat ethnobotanically against anticancer activities.

## 9. Attitudes of Consumers to WEPs

Strong convictions existed regarding the superior ability of wild foods to preserve the health of those who relied on them, primarily among the indigenous people [46]. According to Anbessa [42], most edible wild plants were consumed as extra food instead of daily meals. During food scarcity and starvation periods, when the stocks of stored food crops were gradually diminishing, more than 70% of the edible wild plants were eaten [27]. The general people consume the majority of WEPs as snacks, dietary supplements, or drinks. Therefore, the majority of the local indigenous population views the WEPs as food for starvation or famine on occasion. Of the WEPs, about 73.33% were used as additional food, and the remaining 26.67% were used as meals or normal food [12]. This suggests that WEPs are viewed as famine food by the majority of people.

Eating edible plants found in the wild is associated with lower social classes and is viewed as disrespectful due to ignorance [48]. Similarly, most people in Kayissa Kebele, South Omo Zone, do not drink 10 WEPs until a severe food crisis affects all socioeconomic classes, from the richest to the lowest [139]. According to several Ethiopian investigations, the taboo seems to be avoiding becoming completely depressed to the extent of ingesting WEPs [48]. WEPs are crucial for food security, but their use has been limited by several issues, including cultural illiteracy, the difficulty of gathering, and the fact that they are select foods [43]. In a related study, Tsehay et al. [85] found that while most people in the community understood and believed that WEPs were crucial for filling in food gaps and acting as dietary supplements, some saw the use of WEPs as an indication of poverty and underdevelopment. This severely threatens the conservation and consumption of WEPs. However, Addis, Urga, and Dikasso [51] reported that Ethiopians use wild plants as a food source during both periods of plenty and scarcity. Although households have expressed a desire to keep hunting and gathering wild foods, the amount they could obtain was very small because staple crops have replaced wild foods in the market, and hunting and gathering wild foods is illegal. Wild foods are not culturally acceptable [46]. The research shows that people's perceptions of WEPs as food differ depending on age, gender, economic standing, and where they are perceived as food.

## 10. Conclusion

The present article attempts to review the available information regarding the nutritional contribution, supplementary role, and medicinal value of WEPs in Ethiopia. WEPs have the potential to significantly improve food security by providing alternative sources of affordable and nutritious food with the added advantage of being available all year round. Fruits are the dominant parts of WEPs used for medicinal purposes and consumption. Besides nutritional contributions, WEPs are used as medicine to treat different human diseases and are sold to generate income for rural people. Nutrition policies must promote the utilization of WEPs as part of a strategy to improve food security, nutrition, and livelihoods of rural communities throughout the country. Further investigation of WEPs' bioactive compounds and antinutritional factor contents is needed.

## Figures and Tables

**Table 1:** Widely utilized wild edible fruit, leaves, stem, flower, and shoot of well-known species in different geographic locations of Ethiopia and their mode of consumption.

**Scientific name**	**Habitat**	**Edible parts**	**Modes of consumption**	**Collection month**	**Geographic locations**	**References**
*A. sennii*	Shrub	Flower nectar	Raw	October–December	Chilga District, northwestern Ethiopia	[55]
*A. sennii*	Shrub	Flower	Nectar	May–October	West Gojjam Zone, North Ethiopia	[56]
*A. angustifolius*	Herb	Leaf	Boiled	February and March	Konso wereda, South Ethiopia	[8]
*A. caudatus*	Herb	Young leaves	NR	July and September	Derashe and Kucha districts, South Ethiopia	[10]
*A. caudatus*	Herb	Leaves	Cooked leaves and seeds	March–April and October–November	Soro District of Hadiya Zone, southern Ethiopia	[57]
*A. dubius*	Herb	Leaves	Roasted/cooked leaves	March–April and October–November	Soro District of Hadiya Zone, southern Ethiopia	[57]
*A. hybridus*	Herb	Seed	Porridge	February and March	Konso wereda, South Ethiopia	[8]
*A. gomboczianus*	Herb	Root	Porridge	February and March	Konso wereda, South Ethiopia	[8]
*A. gangetica*	Herb	Leaf	Boiled	February and March	Konso wereda, South Ethiopia	[8]
*B. rotundifolius*	Tree	Seed	Porridge	February and March	Konso wereda, South Ethiopia	[8]
*B. aegyptiaca*	Tree	Fruit	Fresh	October–September	Boosat and Fantalle districts of semiarid East Shewa	[54]
*B. longissima*	Shrub	Flower	Eaten raw	February and March	Konso wereda, South Ethiopia	[8]
*B. pachyloma*	Herb	Leaves	Cooked leaves and shoots	March–April and October–November	Soro District of Hadiya Zone, southern Ethiopia	[57]
*B. edulis* Bull	Herb	Shrub	Prepared/cooked	October–December	Chilga District, northwestern Ethiopia	[55]
*C. spinarum* L.	Shrub	Fruit	Porridge	February and March	Konso wereda, South Ethiopia	[8]
*C. gynandra*	Herb	Leaf	Raw	May–March	Bench-Maji Zone of Southwest Ethiopia	[58]
*C. olitorius*	Herb	Young leaves	NR	July and September	Derashe and Kucha districts, South Ethiopia	[10]
*C. olitorius*	Herb	Leaves	Prepared/cooked	October–December	Chilga District, northwestern Ethiopia	[55]
*C. trilocularis*	Herb	Young leaves	NR	July and September	Derashe and Kucha districts, South Ethiopia	[10]
*C. africana*	Tree	Fruits	NR	July and September	Derashe and Kucha districts, South Ethiopia	[10]
*D. cinerea*	Tree	fruit	Fresh	October–December	Chilga District, northwestern Ethiopia	[55]
*D. praehensilis*	Herb	Tuber	Raw	May–March	Bench-Maji Zone of Southwest Ethiopia	[58]
*D. praehensilis*	Climber	Root	NR	July and September	Derashe and Kucha districts, South Ethiopia	[10]
*D. prahensilis*	Climber	Root	Prepared/cooked	October–December	Chilga District, northwestern Ethiopia	[55]
*D. schimperiana*	Climber	NR	Mature tuber cooked	March–April and October–November	Soro District of Hadiya Zone, southern Ethiopia	[57]
*D. abyssinica*	Tree	Fruit	Fresh	October–December	Chilga District, northwestern Ethiopia	[55]
*D. mespiliformis*	Tree	Fruit	Fresh	October–December	Chilga District, northwestern Ethiopia	[55]
*D. glabra*	Tree	Seed	Porridge	February and March	Konso wereda, South Ethiopia	[8]
*D. abyssinica*	Tree	Fruit	Fresh	October–December	Chilga District, northwestern Ethiopia	[55]
*F. communis*	Herb	Leaf	Fresh leaf	May–October	West Gojjam Zone, North Ethiopia	[56]
*F. sur* Forssk	Tree	Fruit	Fresh	October–December	Chilga District, northwestern Ethiopia	[55]
*F. sycomorus*	Tree	Fruit	Fresh	October–December	Chilga District, northwest Ethiopia	[55]
*F. sycomorus*	Tree	NR	Ripe, raw fruits	March–April and October–November	Soro District of Hadiya Zone, southern Ethiopia	[57]
*F. vasta* Forssk	Tree	Fruit	Fresh	October–December	Chilga District, northwestern Ethiopia	[55]
*G. tenax*	Shrub	Fruit	Fresh fruits	April–June	Boosat and Fantalle districts of semiarid East Shewa	[54]
*J. calyculata* Deflers	Herb	Leaf	Boiled	February and March	Konso wereda, South Ethiopia	[8]
*J. flava*	Herb	Leaf	Boiled	February and March	Konso wereda, South Ethiopia	[8]
*O. spinosa*	Tree	NR	Ripe raw fruits	March–April and October–November	Soro District of Hadiya Zone, southern Ethiopia	[57]
*O. ficus-indica*	Shrub	Fruit	Fresh fruit	May–October	West Gojjam Zone, North Ethiopia	[56]
*P. thonningii*	Tree	NR	Raw and cooked leaves	March–April and October–November	Soro District of Hadiya Zone, southern Ethiopia	[57]
*R. glutinosa*	Tree	Fruit	Fresh ripe	May–October	West Gojjam Zone, North Ethiopia	[56]
*S. nigrum*	Weed	Leaf	Raw	May–March	Bench-Maji Zone of Southwest Ethiopia	[58]
*S. africanus*	Herb	Seed	Dried and prepared	October–December	Chilga District, northwestern Ethiopia	[55]
*S. pyramidalis*	Herb	Seed	Porridge	February and March	Konso wereda, South Ethiopia	[8]
*S. guineense*	Tree	Leaf	Fresh leaf	May–October	West Gojjam Zone, North Ethiopia	[56]
*T. indica*	Tree	Seed	Dried and prepared	October–December	Chilga District, northwestern Ethiopia	[55]
*T. schimperi*	Herb	Leaf	Fresh leaf	May–October	West Gojjam Zone, North Ethiopia	[56]
*T. madagascariense*	Tree	Fruit	Raw	May–March	Bench-Maji Zone of Southwest Ethiopia	[58]
*V. membranacea*	Herb	Leaf/seed	Raw	May–March	Bench-Maji Zone of Southwest Ethiopia	[58]
*W. ugandensis*	Tree	NR	Ripe raw fruits	March–April and October–November	Soro District of Hadiya Zone, southern Ethiopia	[57]
*X. americana*	Shrub	Fruit	Fresh fruits	October–September	Boosat and Fantalle districts of semiarid East Shewa	[54]
*Z. spina*-*christi*	Herb	Fruit	Fresh fruits	October–September	Boosat and Fantalle districts of semiarid East Shewa	[54]

Abbreviation: NR, not reported.

**Table 2:** Proximate composition percentage of dry weight basis (%, dwb) and gross energy (kilocalorie /100 g) of the underutilized wild edible.

**Name of plant species**	**Part of plant**	**Moisture**	**Protein**	**Fat**	**Fiber**	**Ash**	**Carbohydrate**	**Energy**	**References**
*A. ellenbeckii*	Leaves	83.6	27.7	5.0	10.0	13.8	43.5	174.1	[67]
*A. gomboczianusa*	Tuber	84.5	5.8	0.4	4.3	6.0	83.5	333.8	[9]
*A. graecizans*	Leaves	40.8	14.97	8.40	9.70	24.7	1.43	141.2	[61]
*A. graecizans*	Leaves	72.7	28.5	3.9	8.5	22.0	37.1	148.2	[67]
*A. hybridus*	Grain	9.17	23.31	9.83	10.76	6.99	58.29	414.80	[39]
*A. hybridus*	Leaves	7.18	17.63	1.88	6.21	15.22	66.25	352.40	[39]
*B. aegyptiaca*	Leaves	63.5	28.8	2.5	15.5	12.5	40.7	162.6	[67]
*C. argentea*	Leaves	84.1	32.7	2.9	9.8	23.9	30.7	122.9	[67]
*C. grandis*	Leaves	78.5	36.3	3.5	10.1	15.2	34.9	139.6	[67]
*C. grandis*	Leaves	87.3	28.7	5.2	NR	15.9	NR	NR	[9]
*C. trilocularis*	Leaves	83.9	20.4	1.5	11.1	15.4	51.7	206.8	[67]
*D. glabra*	Fruit	28.10	16.00	0.46	2.41	6.79	48.65	253.10	[67]
*E. abyssinica*	Fruit	9.83	3.01	1.46	2.11	4.84	78.27	342.63	[34]
*E. abyssinicum*	Leaves	29.90	17.47	6.87	7.80	4.97	32.99	263.46	[61]
*E. arabicum*	Leaves	8.34	30.15	3.80	21.54	22.75	30.11	275.17	[39]
*E. abyssinicum*	Leaves	8.58	33.63	1.90	15.53	18.02	39.50	309.61	[39]
*F. mucuso*	Fruit	10.64	5.11	3.31	0.93	8.14	71.87	337.71	[34]
*G. Erubescens*	Fruit	10.01	4.22	1.40	1.26	5.23	77.88	341.99	[34]
*H. rueppelii*	Leaves	6.95	13.10	2.67	20.71	12.83	57.64	307.02	[39]
*H. schimperi*	Leaves	6.50	24.04	3.20	17.93	20.02	41.30	290.14	[39]
*J. flava*	Leaves	80.6	32.9	2.7	7.5	25.6	31.3	125.4	[67]
*J. ladanoides*	Leaves	73.4	25.4	2.9	12.5	25.3	33.9	135.5	[67]
*L. hastata*	Leaves	76.9	20.3	5.5	4.9	13.8	45.5	182.0	[9]
*L. intybacea*	Leaves	80.1	24.1	3.7	10.7	21.4	40.1	160.3	[67]
*O. cusindica*	Fruit	12.4	11.70	11	18.80	1.7	44.4	326.4	[61]
*P. laticoronum*	Leaves	90.5	8.1	3.1	15.1	13.2	60.5	242.1	[9]
*P. insipidum*	Leaves	77.0	32.3	3.3	10.9	15.2	38.0	151.9	[9]
*P. quadrifida*	Leaves	90.9	19.6	3.1	15.9	24.6	36.8	147.3	[9]
*R. abyssinicus*	Shoots	39.53	9.53	14.07	34.70	0.9	1.27	169.8	[61]
*R. nervosus*	Shoot	7.99	20.60	1.08	43.77	12.31	30.23	213.05	[39]
*T. foenum-graecum*	Leaves	89.8	28.0	5.8	NR	23.8	NR	NR	[9]
*U. simensis*	Leaves	9.77	30.55	3.29	7.48	26.35	42.11	320.26	[39]
*U. simensis*	Leaves	25	12.30	4.40	10.47	21.30	26.53	194.9	[61]
*X. caffrab*	Fruit	61.2	21.6	23.6	10.4	5.0	39.4	157.5	[9]
*Z. spina-christi*	Fruit	13.10	5.31	1.65	0.71	9.23	70.41	316.05	[34]

Abbreviation: NR, not reported.

**Table 3:** Mineral content dry weight basis (mg/100 g, dwb) of the underutilized wild edible plants in Ethiopia.

**Name of WEPs**	**Part of plant species**	**Calcium**	**Sodium**	**Iron**	**Potassium**	**Zinc**	**Magnesium**	**Reference**
*A. ellenbeckii*	Leaves	1239	NF	16.6	NF	3.1	404	[72]
*A. hybridus*	Grain	55.01	37.99	20.33	14.40	16.84	70.49	[39]
*A. hybridus*	Leaves	59.94	25.53	18.81	34.79	8.35	70.59	[39]
A. graecizans	Leaves	3029	NF	19.3	NF	2.3	2049	[72]
*A. gomboczianus*	Tuber	428	NF	8.72	NF	1.1	109	[9]
*B. aegyptiaca*	Leaves	2487	NF	13.5	NF	1.2	701	[72]
*C. argentea*	Leaves	2207	NF	19.8	NF	2.2	824	[72]
*C. grandis*	Leaves	3064	NF	13.0	NF	2.5	433	[72]
*C. trilocularis*	Leaves	1767	NF	18.6	NF	2.9	175	[72]
*D. glabra*	Fruit	13.88	NF	NF	NF	NF	1.16	[5]
*D. abyssinica*	Fruit	120.18	10.86	2.09	183.36	0.62	5.62	[34]
*E. abyssinicum*	Leaves	49.73	32.86	13.23	43.57	14.25	65.31	[39]
*E. arabicum*	Leaves	44.35	26.49	15.07	32.79	8.76	56.65	[39]
*F. mucuso*	Fruit	190.18	4.88	20.96	165.84	0.62	56.55	[34]
*G. erubescens*	Fruit	98.89	7.21	15.04	107.54	6.23	3.44	[34]
*H. rueppelii*	Leaves	59.05	28.67	22.14	40.77	23.87	62.99	[39]
*H. schimperi*	Leaves	49.37	32.46	27.96	54.30	17.97	65.14	[39]
*J. flava*	Leaves	3419	NF	20.6	NF	2.7	547	[72]
*L. intybacea*	Leaves	2070	NF	22.0	NF	3.1	437	[9]
*L. hastata*	Leaves	1699	NF	14.2	NF	2.0	214	[9]
*M. stenopetala*	Leaves	792.8	NF	2.89	NF	0.53	NF	[72]
*P. laticoronum*	Leaves	1128	NF	13.2	NF	2.4	309	[9]
*P. insipidum*	Leaves	1100	NF	16.3	NF	2.1	183	[9]
*P. quadrifida*	Leaves	2193	NF	20.1	NF	2.9	1094	[9]
*R. nervosus*	Shoot	54.11	30.03	10.51	41.18	11.53	61.82	[39]
*T. foenum-graecum*	Leaves	1038	NF	23.14	NF	1.0	203.5	[9]
*U. simensis*	Leaves	60.14	33.46	16.57	30.58	12.18	72.79	[39]
*X. caffra*	Fruit	180	NF	1.9	NF	1.3	110	[9]
*Z. spina-christi*	Fruit	67.17	6.01	29.13	148.91	8.34	81.15	[34]
*Z. spina-christi*	Leaves	339.5	NF	71.99	NF	2.7	76.3	[72]

Abbreviation: NF = not found.

**Table 4:** Responsible phytochemicals for the antioxidant activities of wild edible plants in Ethiopia.

**Plant**	**Family**	**Plant part**	**Responsible phytochemicals**	**Biological effects**	**References**
*A. hybridus*	Amaranthaceae	Seed	Oxalate, phytate, and tannins	Antioxidant and antinutritional	[79]
*R. nervosus*	Polygonaceae	Shoot	Oxalate, phytate, and tannins	Antioxidant and antinutritional	[79]
*E. arabicum*	Brassicaceae	Leaves	Polyphenol, flavonoids	Antioxidant and antinutritional	[79]
*A. digitata*	Bombacaceae	Fruit, leaf	Phenolic and flavonoid, vitamin C	Proinflammatory	[105]
*A. ellenbeckii*	Passifloraceae	Leaves	Tannins, oxalates, phenolics, micronutrients, amino acids	Ethnobotany	[8]
*A. caudatus*	Amaranthaceae	Leaves, grain	Tocotrienol, tocopherol, cholesterol	Cholesterol-lowering	[106]
*A. dubius*	Amaranthaceae	Leaves	Flavonoid and phenolic compounds	Nutraceutical	[100]
*A. graecizans*	Amaranthaceae	Leaves	Tannins, oxalates, phenolics	Nutraceutical	[100]
*A. hybridus*	Amaranthaceae	Leaves	Polyphenols, flavonoids, flavonols, and proteins	Antioxidant activity	[107]
*A. viridis*	Amaranthaceae	Leaves	Polyphenols, flavonoids, flavonols, and proteins	Nutraceutical	[100]
*A. gomboczianus*	Araceae	Tubers	Carbohydrate phenolics, tannins. Minerals present	Ethnobotany	[8]
*B. aegyptiaca*	Balanitaceae	Fruit/leaves	Phenolics, tannins, and oxalic acid	Antioxidant and nutritional	[8, 108]
*C. africana*	Ulmaceae	Leaves and stems	Phenolics	Medicinal potentials	[109]
*C. anisata*	Rutaceae	Leaves	Leaf phenolic and flavonoids	Antioxidant and acetylcholinesterase inhibitory properties	[110]
*C. argentea*	Amaranthaceae	Leaves	Oxalates, tannins	Ethnopharmacology	[8, 111]
*C. abyssinica*	Cucurbitaceae	Tuber	Phytate, tannin, oxalate, and cyanide	Antinutritional	[112]
*C. grandis*	Cucurbitaceae	Leaves	Phenolics, tannins, and oxalic acid	Antioxidant activity	[113]
*C. aestuans*	Tiliaceae	Leaves	Flavonoids, carbohydrates, saponins, phytosterols, triterpenoids, cardiac glycosides, and tannins	Antioxidant activity	[114]
*C. trilocularis*	Tiliaceae	Leaves	Phenolics, tannins, and oxalic acid; minerals, amino acids	Ethnobotany	[8]
*D. stramonium*	Solanaceae	Leaves	Alkaloids, flavonoids, terpenes, tannins, saponins, iridoids, glycosides, and sterols	Antibacterial and antioxidant activity	[115]
*D. muricata*	Amaranthaceae	Stem, leaf, root	Phenols, tannins, terpenoids, flavonoids, and glycosides	Antidiabetic activity	[116]
*E. capensis*	Meliaceae	Leaves and Twigs	Iridoid, phenolic, and flavonoid contents	Antioxidant and acetylcholinesterase inhibitory properties	[110]
*F. palmata*	Moraceae	Fruit, leaf	Alkaloids, tannins, flavonoids, terpenoids, and cardiac glycosides		[117]
*F. sychomorous*	Moraceae	Flesh with peel and seed	Alkaloids, carbohydrates, flavonoids, saponins, steroids, tannins, phenols, triterpenoids, anthracenosides, anthocyanins, and coumarin	Alpha-glucosidase inhibitory potential	[118]
*L. camara*	Verbenaceae	Leaves	Pentacyclic triterpenoids. Alkaloids, essential oils, iridoid glycosides, phenyl ethanoid, quinine, saponins, steroids, triterpens, sesquiterpenoids, and tannin	Antioxidant activity	[119]
*L. intybacea*	Asteraceae	Leaves	Minerals, tannins, oxalates, and phenolics	Ethnobotany	[8]
*L. hastata*	Asclepiadaceae	Leaves	Flavonoids, proanthocyanidins, alkaloids, and saponins	Alpha-glucosidase inhibitory potential	[120]
*M. kummel*	Sapotaceae	Pulp	Polyphenol and tannic acid	Proinflammatory	[105]
*M. mesozygia*	Moraceae	Stem bark	Flavonoids		[121]
*P. laticoronum*	Asclepiadaceae	Aerial	Phenolics, tannins and oxalic acid, and having micronutrients	Ethnobotany	[8]
*P. oleracea*	Portulacaceae	Leaves	Chlorogenic, caffeic, *p*-coumaric, ferulic, and rosmarinic acids	Antidepressant activities	[122]
*V. paradoxa*	Sapotaceae	Fruit	Phenolic and flavonoids	Alpha-glucosidase inhibitory potential	[118]
*U. simensis*	Urticaceae	Leaves	Polyphenol and tannic acid	Proinflammatory	[8, 105]
*Z. mauritiana*	Rhamnaceae	Fruit	Phenolic and flavonoid	Enzymatic antioxidant activities	[123]
*Z. mucronata*	Rhamnaceae	Leaf	Iridoid, phenolic, and flavonoid	Antioxidant and acetylcholinesterase inhibitory properties	[110]

**Table 5:** Anticancer properties of fruit of wild edible plant in Ethiopia.

**Wild edible plants**	**Family**	**Habitat**	**Geographical location**	**Use of plant extract**	**References**
*L. siceraria*	Combretaceae	Tree	Dega Damot District/Amhara Region	The leaves of the plant are powdered, squeezed, and put on the affected area (wound)	[133]
*D. abyssinica*	Flacourtiaceae	Shrub	Fiche District in Oromia Region	Eating 6–10 fruits per day	[134]
*D. sinica*	Flacourtiaceae	Shrub	Dalle District, Fiche District in Oromia Region	The raw bark of the plant is chewed and swallowed. 6–10 fruits are given to eat	[134, 135]
*L. avolkensii*	Meliaceae	Tree	Bensa in SNNP	The leaves and fruit of the plant chopped and mixed with water and taken orally	[136]
*M. africana*	Myrsinaceae	Shrub	Fiche District in Oromia Region	Dried fruit and leaves of plant powdered and mixed with little water and taken orally	[134]
*P. granatum*	Punicaceae	Tree	Libo Kemke in Amhara Region	Crushed the fruit of the plant and eaten	[130]
*F. angolensis*	Rutaceae	Shrub	NA	The juice made from the fruit of the plant taken orally and applied externally to the affected area	[137]
*M. kummel*	Sapotaceae	Tree	Berbere District in Oromia Region	The plant's dried fruits are crushed and mixed with water before being consumed orally	[138]

## Data Availability

The source of the data and materials used in this study has been duly acknowledged in the manuscript.
